# Mandibuloacral Dysplasia Caused by *LMNA* Mutations and Uniparental Disomy

**DOI:** 10.1155/2014/508231

**Published:** 2014-02-03

**Authors:** Shaochun Bai, Anthony Lozada, Marilyn C. Jones, Harry C. Dietz, Melissa Dempsey, Soma Das

**Affiliations:** ^1^University of Chicago, 5841 South Maryland Avenue, Chicago, IL 60637, USA; ^2^University of California and Children's Hospital of San Diego, San Diego, CA 92123, USA; ^3^Johns Hopkins University School of Medicine and Howard Hughes Medical Institute, Baltimore, MD 21287, USA; ^4^Department of Human Genetics, University of Chicago, 5841 South Maryland Avenue, Chicago, IL 60637, USA

## Abstract

Mandibuloacral dysplasia (MAD) is a rare autosomal recessive disorder characterized by postnatal growth retardation, craniofacial anomalies, skeletal malformations, and mottled cutaneous pigmentation. Hutchinson-Gilford Progeria Syndrome (HGPS) is characterized by the clinical features of accelerated aging in childhood. Both MAD and HGPS can be caused by mutations in the *LMNA* gene. In this study, we describe a 2-year-old boy with overlapping features of MAD and HGPS. Mutation analysis of the *LMNA* gene revealed a homozygous missense change, p.M540T, while only the mother carries the mutation. Uniparental disomy (UPD) analysis for chromosome 1 showed the presence of maternal UPD. Markers in the 1q21.3–q22 region flanking the *LMNA* locus were isodisomic, while markers in the short arm and distal 1q region were heterodisomic. These results suggest that nondisjunction in maternal meiosis followed by loss of the paternal chromosome 1 during trisomy rescue might result in the UPD1 and homozygosity for the p.M540T mutation observed in this patient.

## 1. Introduction

Mandibuloacral dysplasia (MAD) is a rare autosomal recessive disorder characterized by postnatal growth retardation, craniofacial anomalies, skeletal malformations, and mottled cutaneous pigmentation [1, 2]. Hutchinson-Gilford Progeria Syndrome (HGPS) is an autosomal dominant disorder demonstrating varying symptoms including short stature, hair loss, joint degeneration, and atherosclerosis [3]. Pathogenic mutations in the *LMNA* gene on chromosome 1q22 and encoding the Lamin A/C protein have been reported in both MAD and HGPS. To date, the majority of cases of MAD are caused by missense mutations in exons 8–10 of the *LMNA* gene [4, 5] that codes for the LAP2 and emerin-binding domain of the Lamin A/C protein.

Recently, we encountered a two-year-old boy with overlapping features of MAD and HGPS. *LMNA* sequence analysis was performed to determine the genetic cause of his clinical phenotype. With the aim of identifying the molecular etiology of this boy's phenotype, a comprehensive study was performed on the patient and parents.

## 2. Materials and Methods

### 2.1. *LMNA* Sequence Analysis

The 12 coding exons plus exon-intron boundaries of the *LMNA* gene were amplified by polymerase chain reaction (PCR). The purified PCR products were sequenced in both directions using ABI Big Dye terminator mix (Life Technologies, Foster City, CA). Data were analyzed using Mutation Surveyor 3.20 software (SoftGenetics, LLC, PA).

### 2.2. *LMNA* Deletion Analysis by Real-Time Quantitative-PCR

Real-time quantitative-PCR (RT-qPCR) was performed using 3 different primer pairs specific to exon 10 of the *LMNA* gene and detected using Power SYBR Green (Life Technologies) following manufacturer instructions. The relative copy number was calculated based on the standard curve method and compared to *PMP22* gene, which was used as an internal control. A ratio of 0.8–1.2 was indicative of no deletion/duplication.

### 2.3. Microsatellite Analysis

Genotyping of microsatellite markers on chromosomes 1, 6, and 15 was performed on the patient and both parental samples. Microsatellite markers were amplified and separated on an ABI PRISM 3130xl Genetic Analyzer (Life Technologies). The PCR fragments were analyzed using ABI PRISM GeneScan and Genotyper software (Life Technologies).

This study was approved by the University of Chicago Institutional Review Board (IRB protocol number 11-0151).

## 3. Results 

### 3.1. Clinical Phenotype

**Figure 1:**
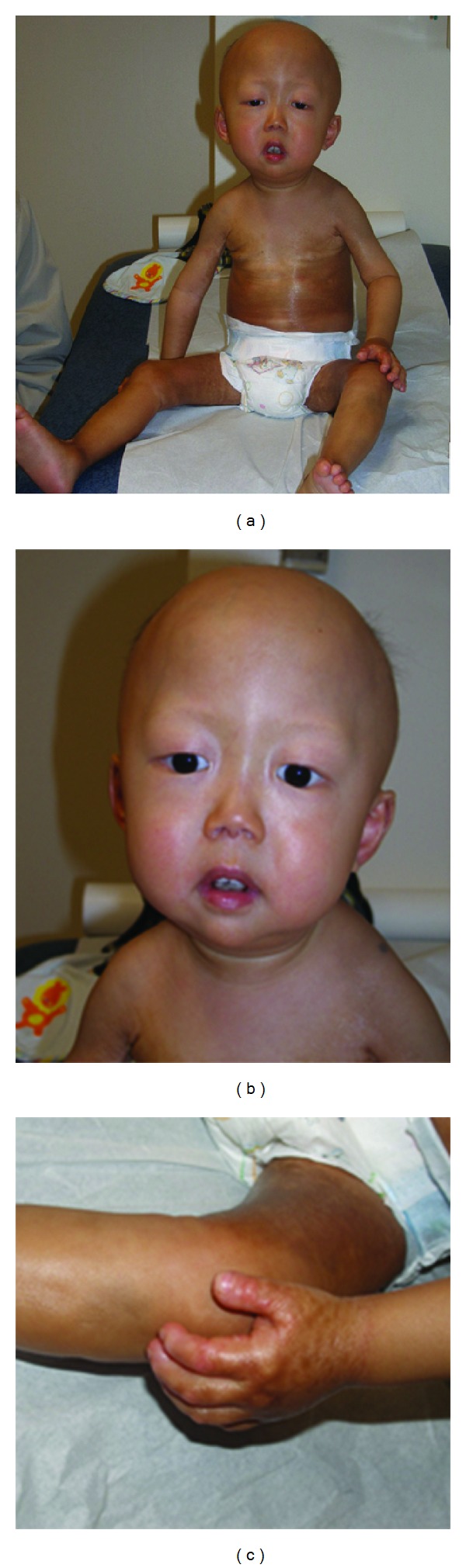
Clinical features of the patient with a homozygous mutation p.M540T. (a) presents proband's hyperpigmented and thickened skin (especially on his truck and thighs) and thin clavicles. (b) depicts patient's prominent cranium and the loss of hair. (c) demonstrates his small contracted hands with bulbous distal tips.

The patient is a two-year-old boy from a nonconsanguineous family of Chinese descent. At 3 months of age, he started presenting with progressive hair loss. Thickening of the skin on his knees developed at 6 months of age followed by progressive joint contractures and hyperpigmentation with sclerosis of the skin. By 1 year of age, his weight was reduced to below the 3rd percentile; he also developed stiffness and blunting of the fingertips. Osteoporosis was noted on radiographs. At 18 months of age, his head circumference, height, and weight were 50th, 25th, and below the 3rd percentiles, respectively. He was cognitively normal with physical restrictions related to joint contractures. He had striking alopecia, prominent scalp veins, limited jaw mobility, and dental crowding. His hands were small and contracted with bulbous distal tips and purplish discoloration over the extensor surfaces. Contractures were present in all major joints. His skin was diffusely thick. At 2 years of age, radiographs showed striking acroosteolysis in the clavicles, hands and feet, wormian bones, and osteopenia. His phenotype shared features of both MAD and HGPS (Figure 1). Molecular genetic testing was requested to make the molecular diagnosis. Over time, the patient suffered continued growth failure with progressive skin thickening and stiffness that was partially relieved by topical pimecrolimus. He had a pathological fracture of his radius at the age of 3. Progressive acro-osteolysis of the jaw resulted in premature dental loss. Stamina has decreased.

### 3.2. Molecular Analysis

**Figure 2:**
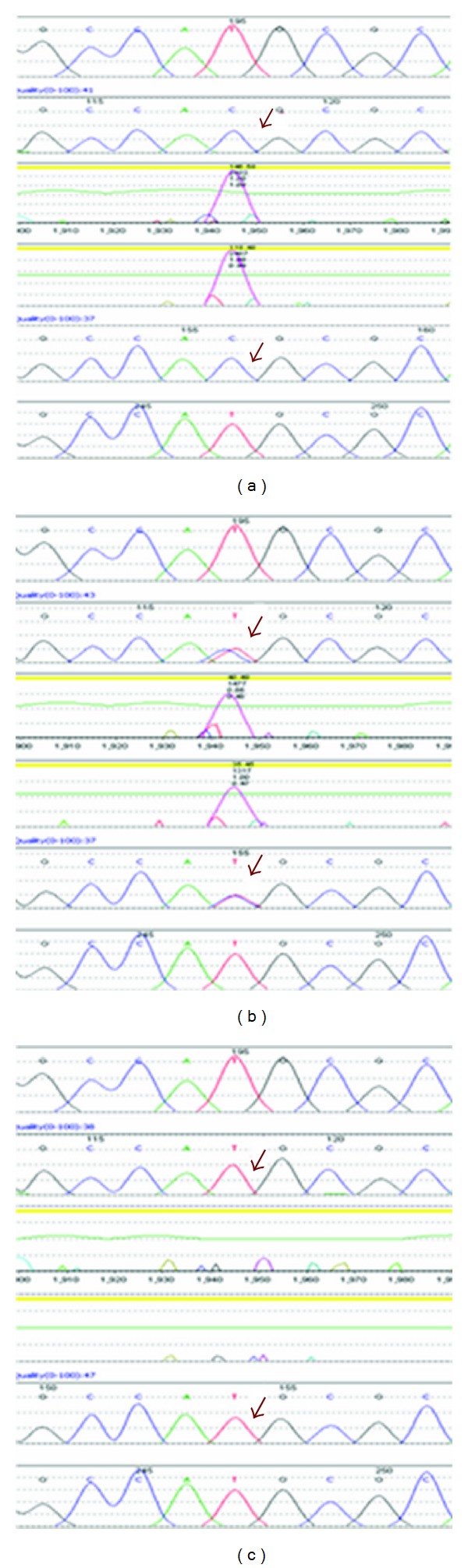
DNA sequencing results. (a) DNA sequencing revealed a homozygous mutation of c.1619T>C, p.M540T in the patient. (b) Mother is a carrier. (c) Father is not a carrier. The c.1619 nucleotide residue is depicted by an arrow in forward and reverse sequence traces for the patient and both parents. The sequence of a normal control sample is shown for each case for comparison.

DNA sequencing revealed a homozygous c.1619T>C, p.M540T mutation in exon 10 of the *LMNA* gene in this patient (Figure 2(a)). Subsequent analysis of the parental samples revealed that the mother was a heterozygous carrier of the same mutation but the father was not (Figures 2(b) and 2(c)). This result was confirmed by repeat PCR/sequence analysis using different sets of PCR primers to rule out the possibility of an SNP interfering with PCR amplification. Nonpaternity was excluded by genotyping of 13 microsatellite markers across chromosomes 6 and 15 (data not shown). The presence of a deletion of one copy of the *LMNA* gene in the patient (which would make the p.M540T mutation appear homozygous) and the patient's father was investigated by RT-qPCR of exon 10 of the *LMNA* gene and two copies of the gene were identified (data not shown).

**Figure 3:**
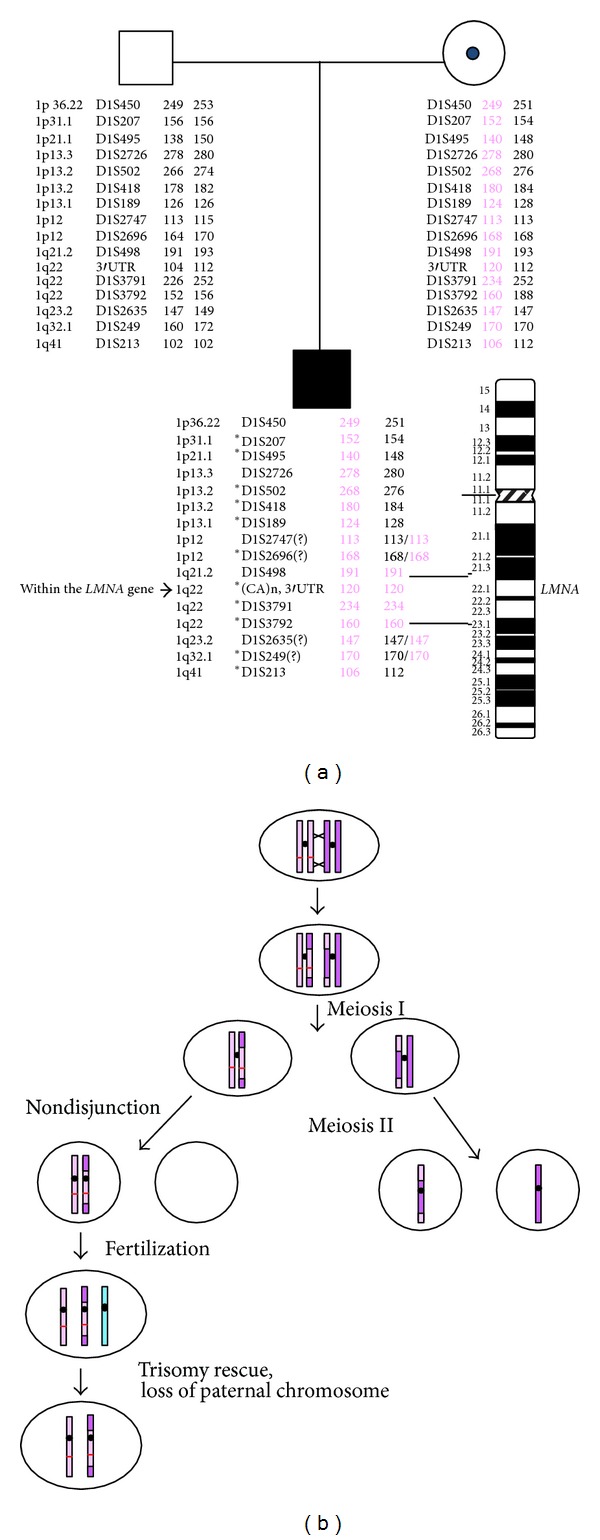
Uniparental disomy analysis of chromosome 1 and schematic representation of the generation of the homozygous p.M540T mutation. (a) Markers around the region of the *LMNA* gene in 1q22 show maternal isodisomy. Allele sizes for each marker are indicated. Informative markers for maternal UPD are denoted with an asterisk (*). Based on the results, one recombination event could have occurred proximal to D1S189 at 1p13.1 and a second recombination event could have occurred proximal to D1S213 at 1q41 resulting in maternal isodisomy for the 1q21-q22 region where the LMNA resides. Due to the uninformative nature of markers D1S2747, D1S2696, D1S2635, and D1S249 in the mother, it is unclear whether these regions are isodisomic or heterodisomic. The uninformative markers are indicated with question marks. (b) Schematic representation of the generation of the homozygous p.M540T mutation in the patient by the process of recombination and nondisjunction in maternal meiosis followed by trisomy rescue. Nondisjunction is shown as occurring in meiosis II in this figure, but it could also have occurred in meiosis I; the microsatellite markers used could not distinguish between these two possibilities. The p.M540T mutation is indicated by the red bar on one of the maternal chromosome 1′s. Paternal chromosome 1 is indicated by the blue colored. *LMNA* gene is indicated by the red bar.

In order to investigate whether uniparental disomy (UPD) involving chromosome 1 may be present in this patient, microsatellite analysis was performed using markers spanning chromosome 1, with increased density in the 1q22 region, where the *LMNA* gene resides. Analysis of 11 informative microsatellite markers showed that the genotype of the patient matched that of the mother with complete absence of the paternal chromosome 1, indicating maternal UPD (Figure 3(a)). Furthermore, the microsatellite data demonstrated that the proband has a minimal region of isodisomy between markers *D1S498 *and *D1S3792*, a 27.2 Mb segment in the 1q21.3–q22 region where *LMNA *is located, with the rest of chromosome 1 being heterodisomic (Figure 3(b)).

## 4. Discussion 

MAD is a rare, autosomal recessive disorder with clinical manifestation involving skin, skeleton, and adipose tissue. The pathogenesis of this rare disorder remains incompletely understood. The majority of MAD patients are caused by point mutations in the *LMNA* gene [6–8]. Patients with overlapping features of MAD and HGPS have also been reported with mutations in the LMNA gene [9–12]. In this study, we report a patient with MAD clinical features, with some overlapping features of HGPS, who presented with a homozygous p.M540T mutation in the *LMNA* gene. This mutation has previously been identified in the compound heterozygous state with another missense mutation in a patient with HGPS in whom atypical pathological findings on fibroblast were observed [13]. The p.M540T mutation affects a conserved region within the C-terminal globular domain of A-type lamins and affects a highly evolutionarily conserved amino acid residue. The observation of this mutation in the homozygous state is the likely cause of the disease phenotype in this patient.

Subsequent analysis of the patient's parents demonstrated only the patient's mother to be a carrier of the p.M540T mutation and the presence of maternal UPD for chromosome 1 as the cause of the homozygosity in the patient. Previous studies have reported UPD as one of the causes leading to the homozygous mutations in several autosomal recessive disorders [14–17]. In fact, UPD involving the *LMNA* gene was identified in some of the first molecularly characterized patients with HGPS [18]. Our patient demonstrated isodisomy for the region encompassing the *LMNA* gene, flanked by heterodisomy. We speculate that during maternal meiosis two recombination events, proximal and distal to the *LMNA* gene, followed by nondisjunction resulted in a gamete with two copies of chromosome 1 both containing the p.M540T mutation. The further loss of paternal chromosome 1 through trisomy rescue after fertilization led to homozygosity of the p.M540T mutation in the patient (Figure 3(b)).

In conclusion, we have identified a patient with MAD and some overlapping features of HGPS in whom a homozygous p.M540T mutation in the *LMNA* gene was identified. The homozygosity of the *LMNA *mutation in this patient was due to maternal UPD of chromosome 1. While UPD involving the *LMNA* gene has previously been identified in patients with HGPS, our patient represents the first case of UPD1 concomitant with *LMNA* mutation in MAD. This observation adds to the growing list of autosomal recessive conditions where UPD contributes to the clinical phenotype.

## Abbreviations

MAD: Mandibuloacral dysplasia
HGPS: Hutchinson-Gilford Progeria Syndrome
UPD: Uniparental disomy.
